# *BRCA1* and Metastasis: Outcome of Defective DNA Repair

**DOI:** 10.3390/cancers14010108

**Published:** 2021-12-27

**Authors:** Rehna Krishnan, Parasvi S. Patel, Razqallah Hakem

**Affiliations:** 1 Princess Margaret Cancer Centre, University Health Network, Toronto, ON M5G 1L7, Canada; rehna.krishnan@uhnresearch.ca (R.K.); parasvi.patel@gmail.com (P.S.P.); 2 Department of Medical Biophysics, University of Toronto, Toronto, ON M5G 1L7, Canada; 3 Department of Laboratory Medicine and Pathobiology, University of Toronto, Toronto, ON M5S 1A8, Canada

**Keywords:** *BRCA1*, *BRCA2*, metastasis, DNA damage, DNA repair

## Abstract

**Simple Summary:**

*BRCA1* has critical functions in accurately repairing double stand breaks in the DNA through a process known as homologous recombination. *BRCA1* also has various functions in other cellular processes that safeguard the genome. Thus, mutations or silencing of this tumor suppressor significantly increases the risk of developing breast, ovarian, and other cancers. Metastasis refers to the spread of cancer to other parts of the body and is the leading cause of cancer-related deaths. In this review, we discuss the mechanisms by which *BRCA1* mutations contribute to the metastatic and aggressive nature of the tumor cells.

**Abstract:**

Heritable mutations in *BRCA1* and *BRCA2* genes are a major risk factor for breast and ovarian cancer. Inherited mutations in *BRCA1* increase the risk of developing breast cancers by up to 72% and ovarian cancers by up to 69%, when compared to individuals with wild-type *BRCA1*. *BRCA1* and *BRCA2* (*BRCA1/2*) are both important for homologous recombination-mediated DNA repair. The link between *BRCA1/2* mutations and high susceptibility to breast cancer is well established. However, the potential impact of *BRCA1* mutation on the individual cell populations within a tumor microenvironment, and its relation to increased aggressiveness of cancer is not well understood. The objective of this review is to provide significant insights into the mechanisms by which *BRCA1* mutations contribute to the metastatic and aggressive nature of the tumor cells.

## 1. Introduction

**Figure 1:**
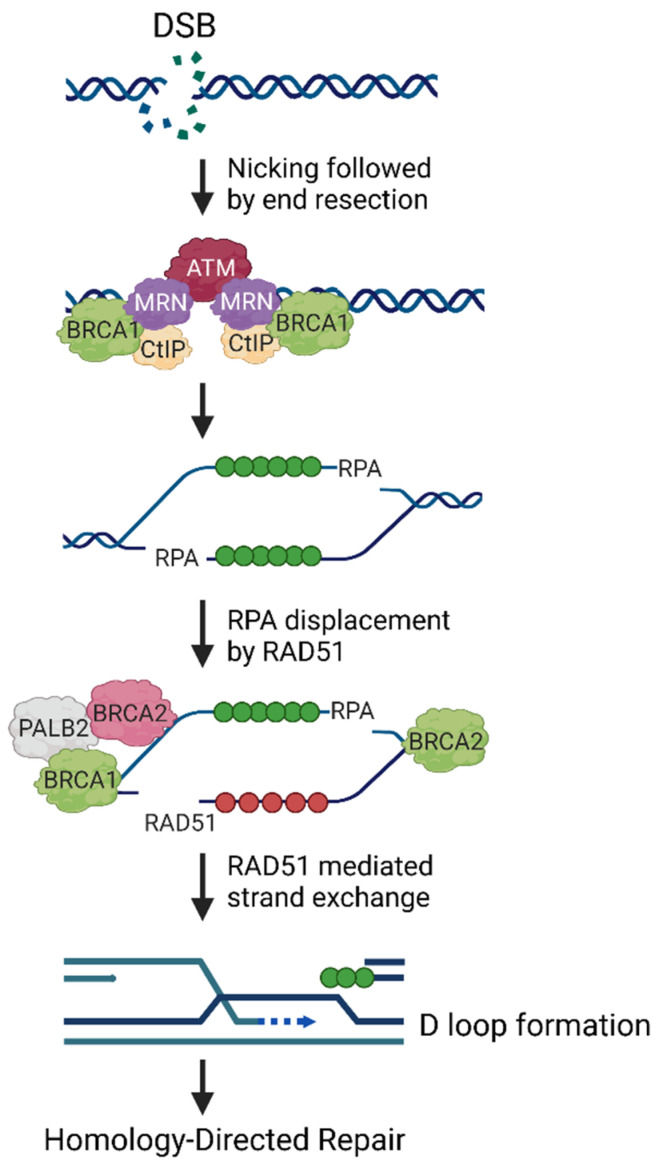
Role of *BRCA1* in DNA double-strand break repair. Homology-directed repair or homologous recombination is an error-free repair pathway of DNA double-strand breaks (DSBs), which occurs primarily during S/G2 cell cycle phases. ATM is a serine/threonine protein kinase that is recruited to and activated at the DNA damage sites by the MRE11, RAD50, and NBS1 (MRN) complex. Upon activation, ATM phosphorylates a large number of substrates to promote DNA repair. DNA end resection is the initial step in determining the DSB repair choice and is controlled by *BRCA1*, MRN, and CtIP. The replication protein A (RPA) binds to single-stranded DNA after the end resection. *BRCA1* promotes the recruitment of PALB2 and *BRCA2* at DNA-damage sites, and the interaction between *BRCA1* and PALB2 is important for the HR repair pathway. *BRCA1*-*PALB2*-*BRCA2* complex leads to the formation of RAD51 filaments on the 3’ single-stranded DNA, followed by the strand invasion into a homologous DNA, initiating the formation of displacement loops (D-Loops), which leads to the resolution of DSBs. Created with Biorender.com.

Breast cancer susceptibility gene 1 (*BRCA1*) encodes the tumor suppressor *BRCA1*, which was first linked to hereditary breast and ovarian cancer in the early 1990s [1]. Compared to the lifetime risk of developing breast (12.9%) and ovarian cancer (2.7%) in the general population, female carriers of pathogenic *BRCA1* mutations are at a significantly higher risk of developing these cancers [2,3]. By age 70, *BRCA1* mutations increase the cumulative risk of developing breast cancer to 47–66% and ovarian cancer to 35–46% [4]. *BRCA1* mutations frequently give rise to the aggressive, higher-grade, triple-negative breast cancer subtype [5]. Given the critical role of *BRCA1* in the repair of DNA double-strand breaks via the error-free homologous recombination (HR) pathway and its additional roles in other cellular processes that safeguard genomic integrity, it is not surprising that mutations in this tumor suppressor gene considerably increase cancer risk (Figure 1) [6,7]. Approximately 5% of breast and 15% of ovarian cancer cases were previously thought to arise due to mutations in *BRCA1/2*; however, HRDetect, a recently proposed weighted model which predicts *BRCA1/2* deficiency, has estimated that up to 22% of breast tumors may carry mutations in these genes [8]. HRDetect also identified HR-deficiency in 69% of triple-negative breast cancers, which are more aggressive, associate with poor prognosis, and present a higher risk of recurrence [9]. Therapeutic strategies that benefit cancer patients with *BRCA1/2*-mutant tumors have shown promising results in targeting tumors with mutations in other genes necessary for HR [10]. Recently, the genome-wide mutational scar-based pan-cancer Classifier of HOmologous Recombination Deficiency (CHORD) estimated that 6% of tumors are HR-deficient with some cancers exhibiting greater prevalence of HR-deficiency (breast: 30%, ovary: 52%) [11]. Thus, treatment strategies that eliminate *BRCA1/2*-mutant tumors are likely to have a substantial impact on various cancer types and reducing the global cancer burden. Though Poly (ADP-ribose) polymerase (PARP) inhibition is at the forefront of *BRCA1/2*-mutant breast and ovarian cancer therapy, many new exciting targets such as POLQ, RAD52, FANCD2, FEN1, APEX2, and RNF168, appear to have therapeutic potential in pre-clinical studies [12,13,14,15,16]. These are reviewed in detail elsewhere [17].

While great strides have been made in identifying synthetic lethal interactors of *BRCA1*, cancer metastasis remains the leading cause of death for all cancers [18]. It is estimated that metastasis is responsible for 60–90% of cancer-related deaths [18,19]. A recent study has also reported that germline *BRCA1* mutations in breast cancer patients appear to be associated with an increased risk of brain metastasis even when accounting for other confounding factors, such as age and stage [20]. In this review, we will discuss the role of *BRCA1* in genomic integrity and cancer metastasis, specifically focusing on *BRCA1* function in epithelial to mesenchymal transition (EMT), cell adhesion, cell invasion, tumor neovascularization, and tumor microenvironment. Understanding cancer cell behavior and associated tissue alterations that arise as a result of loss of *BRCA1* function may help improve current therapeutic strategies employed to treat *BRCA1* mutant cancers.

## 2. *BRCA1* Structure and Function in DNA Double-Strand Break Repair

A plethora of evidence now exists implicating *BRCA1* in DNA double-strand break (DSB) repair and genome stability. *BRCA1* is comprised of several functional domains that interact with different proteins [21]. The Really Interesting New Gene (RING) domain at the N-terminus of *BRCA1* is crucial for its heterodimerization with *BRCA1*-associated RING domain 1 (BARD1) [22]. *BRCA1*-BARD1 interaction has been shown to be important for *BRCA1* stability [21,23]. In addition, *BRCA1*, a RING E3 ubiquitin ligase, is known to ubiquitylate several factors, including histones H2A and H2AX, RNA polymerase II, Estrogen receptor (ER) α, and Hippo signaling protein NF2, among others [24,25]. Though pathogenic mutations in the RING domain are common, it is unclear whether the E3 ligase activity of *BRCA1* is important for tumor suppression. *BRCA1*-BARD1 heterodimer also interacts with the RAD51, a recombinase essential for HR [26]. The central part of *BRCA1* consists of exons 11–13, which codes for more than 60% of the *BRCA1* protein [21]. This region contains the coiled coil (CC) domain, which bridges the interaction of *BRCA1* with Partner and Localizer of *BRCA2* (PALB2), and is indispensable for RAD51 loading and strand invasion during HR [27,28]. The C-terminus of *BRCA1* comprises two copies of the BRCT domain. The tandem BRCT domain is important for establishing the phospho-dependent interaction between *BRCA1* and ABRAXAS, *BRCA1*-interacting helicase 1 (BRIP1), and C-terminal binding protein interacting protein (CtIP) [21,29]. Though it was originally identified in *BRCA1*, the BRCT domain has been identified in a myriad of proteins, most of which have been implicated in checkpoint signaling and DNA repair [30]. The ability of *BRCA1* to recognize different binding partner affects its recruitment and function in DNA damage repair. The tandem BRCT domains are crucial for *BRCA1* function in HR, as they are important for the formation of the *BRCA1*-A, *BRCA1*-B, and the *BRCA1*-C complex (reviewed in detail elsewhere) [7,31]. *BRCA1* BRCT domains are also where clinically relevant mutations are frequently observed; these mutations disrupt the binding surface of the BRCT domain to phosphorylated peptides [32]. In addition, mutations are also common in exon 11, the largest exon, of *BRCA1* [21]. The location of the mutation strongly determines cancer risk and the response of the respective *BRCA1*-mutant tumor or cell line to chemotherapeutics, including PARP inhibitors (PARPi) and cisplatin [33,34]. In addition to its function in HR (Figure 1), *BRCA1* also has important functions in protecting the genome and repairing DNA lesions though various cellular processes. *BRCA1* has been implicated in protecting stalled replication forks, regulating transcription, regulating R-loops, modulating the chromatin, regulating cell cycle, enforcing checkpoints, maintaining telomeres, and in transcription-coupled repair [17,35,36,37]. Most recently, Hatchi and colleagues reported that a species of single-stranded DNA damage-associated small RNAs (sdRNA), generated by a *BRCA1*-RNAi complex, promote DNA repair mediated by PALB2-RAD52 complex at R-loop-forming transcriptional termination pause sites [38].

## 3. *BRCA1* in Cancer Initiation and Metastasis

Mechanisms governing the initiation of *BRCA1*-associated cancers remain elusive. Given the indispensable function of *BRCA1* in maintaining genome stability, it is thought that DNA damage associated with *BRCA1* loss results in random mutations in the genome, which inactivate tumor suppressor genes, such as TP53 or activate oncogenes such as MYC, which, through natural selection, promote tumor formation and metastasis [28,39,40,41]. It should be noted that hormonally driven growth during each menstrual cycle produces reactive oxygen species (ROS), which cause oxidative DNA damage leading to DNA lesions, a subset of which produce replication stress requiring repair through HR via *BRCA1* [42]. It is also widely believed that long-term and repeated exposure to ROS and estrogen metabolites also contribute to breast cancer risk [43]. In addition, mutations in the tumor suppressor *TP53* occur in virtually all *BRCA1*-mutant cancers and are essential for tumor survival. In a recent study, which examined the spectrum of *TP53* mutations in *BRCA1/2* associated high-grade serous ovarian cancer identified *TP53* mutations in 96% of *BRCA1*-mutant tumors [44]. This suggests that *BRCA1* mutations are not the only determinant of breast tumorigenesis. It is likely that defective HR, compromised genomic integrity, repeated exposure to hormonally driven ROS, and acquisition of additional mutations, all contribute to breast cancer initiation associated with mutations or loss of function of *BRCA1*.

**Figure 2:**
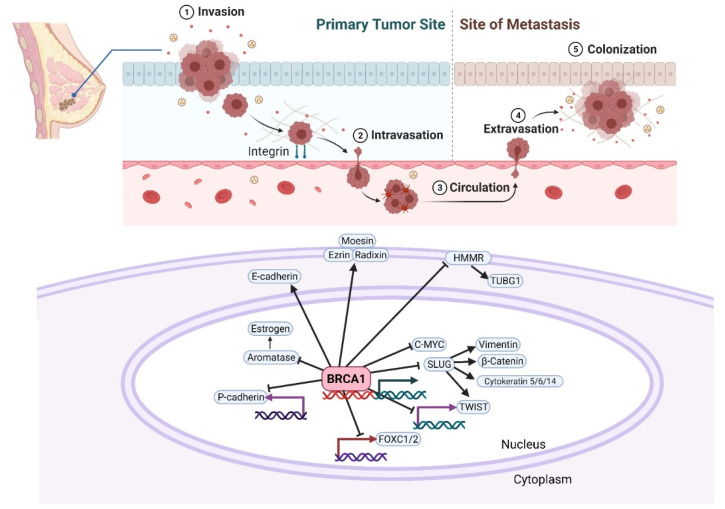
A schematic of the possible effects of *BRCA1* on cancer initiation and metastasis. The different stages leading to metastasis are indicated. Several reports have directly or indirectly implicated *BRCA1* in the regulation of EMT-promoting factors. *BRCA1* mutations in breast cancer cells lead to enhanced EMT phenotype by upregulating key transcription factors like FOXC1/2, TWIST, and SLUG, and by modulating the expression of P-cadherin, cytoskeletal markers, Vimentin, and β-catenin. Breast cancer-associated mutations at the amino-terminus of *BRCA1* abolish its ubiquitin ligase activity and abrogate its intracellular colocalization with ERM (Ezrin-Radixin-Moesin) and F-actin, leading to spontaneous motility of human breast cancer cells. Created with Biorender.com.

While metastasis is estimated to account for 60–90% of cancer-related deaths, it remains one of the most poorly understood components of cancer. Metastasis is a complex process comprised of the following steps: (1) local infiltration of cancer cells into adjacent tissue, (2) transendothelial migration of cancer cells into blood vessels (intravasation), (3) survival in the circulatory system, (4) arrest at distant organ sites, (5) extravasation into the parenchyma of distant organs, and finally, (6) survival and proliferation at metastatic sites (Figure 2) [45]. Though little is known about the function of *BRCA1* in metastasis, several studies have shown increased frequency of metastasis in carriers of *BRCA1* mutations [20,46,47]. Zavitsanos et al. performed a matched pair analysis of breast cancer patients with and without mutations in *BRCA1*/*2* and found that germline *BRCA1* mutations in breast cancer patients associated with increased risk of brain metastasis, even when accounting for other confounding factors, such as age, stage, and expression of hormone receptors ER and human epidermal growth factor receptor 2 (HER2) [20]. Song et al. evaluated the patterns of metastasis in breast cancers associated with *BRCA1/2* mutations. [48]. Lung and distant lymph node metastases were frequently observed in *BRCA1-*mutation carriers, whereas bone metastases were frequently observed in *BRCA2*-mutation carriers [48]. Though this study reported that central nervous system (CNS) metastases were observed at comparable frequencies in both *BRCA1*- and *BRCA2*-mutation carriers, when adjusting for breast cancer subtypes, a significant association with CNS metastases was primarily observed in *BRCA2* mutation carriers. In another study Ratner et al. assessed whether *BRCA1/2* mutations in ovarian cancer increased the risk of brain metastases. This study demonstrated that ovarian cancer patients with mutated *BRCA1/2* had a four-fold increased risk of developing brain metastases and were diagnosed with brain metastases approximately 8 months earlier than patients with wild-type *BRCA1/2* [46]. Given the increased evidence demonstrating that *BRCA1* (and *BRCA2*) mutations greatly influence the course of BC progression and the risk of metastasis, it is critical to understand the function of this protein in the multistep metastasis process.

## 4. *BRCA1* and Epithelial to Mesenchymal Transition

Epithelial to mesenchymal transition (EMT) is an intricate developmental program that enables cancer cells to suppress their epithelial features and change into mesenchymal features that allow them to become mobile and migrate to other sites [49]. In tumors with *BRCA1* mutations, the expression of several key EMT factors is altered. These include transcription factors, cell-surface proteins, cytoskeletal markers, and proteins involved in apicobasal polarity [50]. Though *BRCA1* is not a direct transcriptional repressor of SLUG, a key EMT transcription factor, SLUG is aberrantly expressed in breast tumors with *BRCA1* mutations [51]. In addition, *BRCA1* mutations appear to increase SLUG protein stability [51]. Another study revealed that increased SLUG expression in basal-like breast cancer occurs through β-Catenin-mediated Wnt signaling [52]. *BRCA1* has also been directly implicated in suppressing EMT via inhibiting the upregulation of key EMT transcription factors, TWIST and FOXC1/2. *BRCA1* has been shown to directly bind to the *TWIST* promoter, suppressing its activity and inhibiting EMT in mammary tumor cells [53]. Like TWIST, FOXC1/2 expression is upregulated in *BRCA1*-mutant cancer cells. Mechanistically, *BRCA1*, alongside GATA3, co-repress FOXC1/2 expression by binding to the GATA3 binding site in the promoter region of these genes [54]. Compared to tumors with wild-type *BRCA1*, *BRCA1*-mutated tumors exhibit altered expression of a number of cell-surface proteins [50]. For instance, compared to *BRCA1*-mutant cells, immortalized human mammary epithelial cells with restored *BRCA1* expression exhibit upregulation of E-cadherin and inhibition of P-cadherin, cell adhesion molecules, which are important regulators of cell motility and invasion [55]. Thus, in *BRCA1*-mutant breast cancer cells, P-cadherin expression is increased, contributing to the invasive and metastatic potential of these cancer cells [55]. *BRCA1*-mutated tumors also have upregulated expression of cytoskeletal markers, Vimentin, β-Catenin, Cytokeratin 5/6/14, and downregulated expression of other cytokeratins, which play a critical role in EMT [50,51]. Furthermore, Hyaluronan Mediated Motility Receptor (*HMMR*), a low penetrance breast cancer susceptibility gene important for regulating apicobasal polarity, is upregulated in BRCA1-deficient tumors [56]. Mechanistically, it was demonstrated that *BRCA1* targets HMMR protein for proteasomal degradation. Additionally, the level of HMMR is higher in cell lines derived from *BRCA1* mutant carriers [56,57]. Thus, loss of *BRCA1* leads to HMMR overexpression resulting in loss of polarity through accumulation of the microtubule associated factor TUBG1 [57]. While many mechanisms remain elusive, taken together, it is evident that *BRCA1* has a crucial function, both directly and indirectly, in regulating EMT in breast cancer cells.

## 5. Role of *BRCA1* in Cell Motility and Adhesion

Metastasis is facilitated by cell–cell interactions between tumor cells and the endothelium, and these interactions determine the extent of spread. Direct tumor cell interaction with platelets and leukocytes contributes to cell adhesion and extravasation, leading to the development of metastatic lesions. Recently published reports suggest *BRCA1* involvement in cell migration and adhesion. Expression of targets important for metastasis such as E-cadherin, P-cadherin, caveolin-1 and inhibitor of differentiation-1 (ID1), were found altered upon restoring the expression of the full-length *BRCA1* in *BRCA1* mutant mammary epithelial cell lines [55]. Another study using *BRCA1* proficient (MCF7) and *BRCA1* mutant (HCC1937) breast cancer cell lines reported a role for *BRCA1* in in vitro breast cancer cell spreading, mobility and wound healing [58]. Furthermore, this study identified interactions of *BRCA1* with Ezrin, Radixin, and Moesin, members of the ERM (ezrin-radixin-moesin) family of proteins that crosslink actin filaments with plasma membrane [58]. Overexpression of a truncated form of *BRCA1* lacking its N-terminus ubiquitin ligase domain was found to disrupt endogenous interaction of *BRCA1* with ERM and increase spontaneous motility of human breast cancer cells. These data suggest that *BRCA1* might suppress cell motility by regulating ERM proteins through its ubiquitin ligase activity [58].

Global proteomic analysis of *BRCA1*-deficient ovarian tumor specimens has identified that loss of *BRCA1* associates with altered expression of several factors involved in regulating cell mobility and adhesion, and has a strong association with aggressive ovarian cancers [58]. This proteomic study identified seven candidate proteins with at least 1.5 fold change of expression; six of these proteins (Calpain-1 catalytic subunit (CAPN1), 14-3-3, protein phosphatase 2A (PP2A), macrophage capping protein (CAPG), non-erythrocytic spectrin beta-chain (SPTBN1), profilin 1 (PFN1)) have been demonstrated to either directly or indirectly modulate actin cytoskeleton and cell adhesion [59]. Five candidate proteins from the aforementioned study exhibited differential expression association with both *BRCA1* status and advanced ovarian cancer stage. CAPG and CAPN1 were found to be overexpressed in advanced stages of *BRCA1*-mutant ovarian cancers while PFN1, CFN1, and 14-3-3 were shown to be downregulated. This study also demonstrated that *BRCA1* deficiency in ovarian cancer is associated with changes in the expression of several cytoskeleton and cell adhesion regulatory proteins [59]. However, additional studies are needed to elucidate the mechanisms by which *BRCA1* controls the expression of several major regulators of actin cytoskeleton and cell adhesion, and to uncover the driving forces underlying migration and metastatic phenotypes of *BRCA1*-mutated cancer cells.

## 6. Effect of *BRCA1* on Tumor Microenvironment

The tumor microenvironment (TME) composed of non-cancerous cells plays a major role in the regulation of cancer cell growth and metastasis and has been shown to impact the outcome of the therapy [60,61]. Stromal cells are among the critical components of the tumor microenvironment and their heterogeneous nature depends on the randomly generated mutations within the tumor cells [62]. The impact of *BRCA1* mutations on TME is less well understood. Heterozygous *BRCA1* mutations in mammary tissue microenvironment might create a pro-tumorigenic niche, which may significantly contribute to breast cancer development in carriers of germline *BRCA1* mutations.

Loss of *BRCA1* in mammary epithelial cells have been shown to affect stromal cells in the TME, which in turn enhance the metastatic potential of BRCA1-deficient breast tumors [63,64]. The existence of a complex paracrine loop between tumors and surrounding adipose stromal cells (ASCs) has been hypothesized. Ghosh et al. have reported that *BRCA1* suppresses the transcriptional activity of the breast cancer-associated aromatase promoter in normal ASCs, thus lending further support to the notion that elevated synthesis of estrogen within tumor adipose tissue contributes to the growth of postmenopausal breast cancer [64]. Reduced expression or activity of *BRCA1* due to mutations or epigenetic silencing might result in the reactivation of the aromatase gene, which might lead to abnormal estrogen synthesis and thereby promote breast and ovarian cancer development. Additionally, factors such as interleukin 6 (IL-6) and prostaglandin E2 released by the tumor cells stimulate aromatase expression in *BRCA1*-mutated stromal adipose cells which further enhances estrogen dependent growth of these tumor cells [64,65].

In another study, Weber et al. analyzed whole genome sequencing in a cohort of breast tumors with and without *BRCA1/2* mutations to determine the extent of genomic instability in the malignant breast epithelium and in the tumor stroma [66]. In the case of hereditary *BRCA1/2*-related breast cancers, the frequency of loss of heterozygosity or allelic imbalance (LOH/AI) was found to be approximately equal in the mammary epithelium (59.7%) and the adjacent stroma (66.2%), whereas a higher frequency of LOH/AI was observed in mammary epithelium (36.7%) compared to the stromal compartment (28.4%) in sporadic breast cancers [66]. These results suggest that in patients with *BRCA1/2*-mutated breast cancers, the level of genomic instability in the stroma is equal to that in the epithelium, which could potentially drive breast cancer pathogenesis [66].

BRCA1-IRIS (also known as IRIS; in-frame reading of *BRCA1* intron 11 splice variant) is a variant produced by the alternative splicing of *BRCA1* mRNA and it was reported to have oncogenic functions [67]. It has been proposed that the interactions between *BRCA1*-IRIS overexpressing cells and mesenchymal stem cells (MSCs) result in faster growing metastatic triple-negative breast cancers (TNBC). IL-1β secreted by *BRCA1*-IRIS overexpressing TNBC cells attracts MSCs to the microenvironment and initiates a signaling pathway resulting in C-X-C motif chemokine ligand 1 (CXCL1) secretion by MSCs. This secretion of CXCL1 in turn activates *BRCA1*-IRIS-overexpressing TNBC cells and leads to the secretion of vascular endothelial growth factor (VEGF) and C-C motif chemokine ligand 2 (CCL2). These secreted chemokines attract tumor-associated macrophages (TAMs) and endothelial cells (ECs) to the niche. The metastatic precursors are generated with the help of cytokines (IL-8 and S100A8) secreted from TAMs and ECs in co-operation with CXCL1. This study shows an interesting concept that some tumor types with *BRCA1* alternative splice variants have intrinsic ability to promote their own invasiveness [68]. Together, regulation of *BRCA1* expression and functions in both epithelial and nonepithelial cells within the tumor microenvironment may be important for *BRCA1*-associated tumorigenesis and invasiveness.

## 7. *BRCA1* and Tumor Neovascularization

Several studies have reported that the crosstalk between *BRCA1*-deficient tumor cells and adjacent stromal cells could promote cell survival and migration [66]. The growth and progression of tumors are accompanied by vascularization within the tumors in a process called angiogenesis. Hypoxia-inducible factor 1-*alpha* (HIF1α) levels are elevated in cancer cells during hypoxia and HIF1α dimerizes with HIF1β to activate target genes like VEGF [69,70]. *BRCA1* can modulate tumor growth through its transcriptional regulation of angiogenic factors and the stability of HIF1α. Upregulated expression of HIF1α and VEGF have been observed in *BRCA1/2*-mutated hereditary breast cancer when compared to sporadic breast cancer [71].

Earlier studies have demonstrated that *BRCA1* inhibits the estrogen receptor-signaling pathway through directly binding to it [72]. The interaction of *BRCA1* and estrogen receptor α (ER-α) was observed in MCF-10A breast epithelial cells and MCF-7 breast cancer cells. The stimulation of breast cancer cells with estrogen disrupts the endogenous complex of *BRCA1*-ER-α. Moreover, *BRCA1* and ER-α modulate the expression and secretion of VEGF in breast cancer cells [73]. Interestingly, mutated forms of *BRCA1*, which are overexpressed in familial breast cancers failed to interact with ER-α and did not significantly affect the expression of VEGF [73]. In another study, Danza et al. evaluated the levels of angiogenic axis angiopoetin-1 (Ang-1) and angiopoetin-2 (Ang-2) in familial breast cancer and analyzed its relationship with *BRCA1/2* status [74]. Higher levels of Ang-1 and Ang-2 were observed in patients with *BRCA1/2* mutations. It has been proposed that VEGF, along with Ang-1 and Ang-2, might stimulate the neovascularization in *BRCA1/2*-mutated cancers [74].

*BRCA1* through its interaction with CtIP (CtBP-interacting protein) and ZBRK1 (Zinc finger and *BRCA1*-interacting protein with KRAB domain 1) forms a transcriptional repression complex of Ang-1 by binding to *Ang-1* promoter via its ZBRK1 recognition site [75]. Disruption of the complex upregulates Ang-1 levels which lead to tumor neovascularization. Consistent with the above data, elevated expression of Ang-1 was observed in the mammary tumors from Brca1-deficient mice with significant vascular growth [75]. Given the ability of microRNAs to regulate gene expression, Danza et al. highlighted the impact of miRNAs deregulation on the neovascularization within familial breast cancer [76]. MicroRNA 578 (miR-578) and microRNA 578 (miR-573) were found to be involved in *BRCA1/2*-mutation-related angiogenesis by affecting VEGF, focal adhesion kinase (FAK), and HIF-1-signaling pathways [76]. Taken together, the current data suggests the involvement of *BRCA1/2* in neovascularization and cancer progression.

## 8. *BRCA1* and Cell Invasiveness

The aggressive behavior of *BRCA1*-deficient tumors could be attributed to the random mutations that occur in the genome of these cells due to HR deficiency, which can result in the activation of oncogenes or inactivation of tumor suppressors. Alterations in the tumor microenvironment might be among the important mechanisms underlying the growth and progression of *BRCA1*-deficient tumors. It has been shown that *BRCA1* mutations affect the phenotype of adipose stem cells and induce cell invasiveness [63]. Defective DNA repair pathways in *BRCA1*-mutated adipose stem cells result in the accumulation of DNA damage, thereby activating the ATM pathway and DNA damage response. Higher levels of CDKN1A (P21) due to ATM activation in cells induce senescence and secretion of inflammatory cytokines like IL-6 and IL-8, which promote breast tumor cell proliferation and invasion [63].

In another study, *BRCA1* knockdown in human hTERT-immortalized fibroblasts displayed an elevated rate of growth and invasion [77]. *BRCA1*-depleted fibroblasts expressed significantly elevated levels of autophagy and mitophagy markers and exhibited increased levels of HIF-1α. Elevated levels of ketone bodies in *BRCA1*-depleted fibroblasts are consistent with mitochondrial dysfunction. Xenograft studies demonstrated two-fold increase in tumor growth when the human MDA-MB-231 breast cancer cells were co-injected alongside *BRCA1* knocked-down fibroblasts into nude mice, thus, demonstrating the potential effects of *BRCA1*-deficient tumor stroma on breast cancer growth and invasion [77].

Germline mutations within the tumor or in adjacent stroma might create an environment that promotes the growth of premalignant cells. Russo et al. demonstrated that *BRCA1* and related genes might regulate the epithelial-stroma interaction, thereby regulating lobular development of the breast [78]. Their data suggest that the breast tissue architecture with denser and fibrotic stroma in women with invasive or familial breast cancer is different from the breast tissue of women who underwent reduction mammoplasty or prophylactic subcutaneous mastectomy after genetic counseling [78]. Further studies are warranted to analyze the extent at which germline mutations affect cancer growth, invasion, and clinical outcome.

## 9. Therapeutic Strategies and Management for *BRCA1/2*-Associated Metastatic Breast Cancer

Mutations in *BRCA1* or *BRCA2* genes account for the majority of hereditary breast and ovarian cancers [79]. The penetrance of breast cancer for all *BRCA1/2-*mutation carriers that have no first-degree relative with breast cancer was 60.4% by age 80 and 63.3% for those with at least one first-degree relative with breast cancer [80]. The clinical management of breast cancer should be the same for both groups of patients, but family history should be taken into account during diagnosis and genetic counseling.

The most effective breast cancer prevention and management for *BRCA1/2*-mutation carriers is surgical prevention. Although it is invasive and risky, preventive surgery remains an important step in the cancer management of high-risk individuals. Prophylactic mastectomy is one of the most effective ways to prevent breast cancer development in carriers of *BRCA1/2* mutations [81]. Patients who had bilateral prophylactic mastectomy had a significantly reduced risk of breast cancer development when compared to *BRCA1/2*-mutation carriers with two intact breasts. The risk of ovarian, fallopian tube, and peritoneal cancer was reduced by 80% in *BRCA1/2*-mutation carriers who had undergone preventive bilateral salpingo-oophorectomy (removal of ovaries and fallopian tubes), and was also associated with 77% reduction in all-cause mortality [82,83].

Recent studies have provided sufficient evidence to support the role of chemoprevention agents in high-risk breast cancer patients. Chemoprevention agents include selective estrogen receptor modulators (SERM), such as tamoxifen or aromatase inhibitors (e.g., exemestane) [84]. Tamoxifen is employed as an adjuvant hormonal therapy in ER-positive breast cancer in both pre- and postmenopausal women. Raloxifene, another SERM, is approved only for the treatment of breast cancer in postmenopausal women [85]. Results from two large studies (NSABP-P1 and IBIS-1) showed that tamoxifen treatment reduced the incidence of breast cancer by 40% [86,87]. Interestingly, the treatment was shown to prevent contralateral breast cancer by 50% and demonstrated a 44% risk reduction of developing a second breast cancer in both *BRCA1* and *BRCA2* WT and mutant conditions [88,89]. Despite significant reduction in breast cancer incidence in pre-menopausal women, the side effects of tamoxifen treatment include increased risk of endometrial cancer and pulmonary embolism in post-menopausal women [90]. The availability of safe and effective drugs may significantly change the rate of high-risk women opting for non-invasive preventive treatments.

Effective development of breast cancer therapeutics requires a full understanding of the mechanisms that drive survival of aggressive breast cancer cells. As *BRCA1* and *BRCA2* gene products are involved in homologous recombination, recent advances in therapeutic strategies, which increase sensitivity of *BRCA1/2*-deficient tumors, have provided novel targets for improved treatment of cancers associated with mutations or the loss of expression of these genes [17,91]. *BRCA1/2*-mutant tumors display exquisite sensitivity to platinum salts such as cisplatin and carboplatin, which act as DNA cross-linking agents [91]. Targeting PARP has emerged as a novel therapeutic strategy utilizing the synthetic lethal interaction between PARP and *BRCA1/2* mutations [17,89]. The mechanism of this lethal interaction is associated with the accumulation of DNA double-strand breaks caused by PARP trapping and inhibition [17,92,93]. The treatment of HR defective cancer cells with PARP inhibitors (PARPi) results in persistent DNA double-strand breaks, which lead to cell death [92,93]. A recent report has suggested that PARP inhibitor Olaparib treatment induces changes in the tumor microenvironment of *BRCA1*-mutated TNBC cells and induces CD8^+^ T cell infiltration and activation in vivo [94]. It was proposed that the activation of c-GAS-STING pathway results from the cross talk between PARP inhibition and tumor microenvironment [94]. Several PARPi (e.g., Olaparib, Rucaparib, Talazoparib, and Niraparib) have been approved as monotherapy for either breast, ovarian or both cancers associated with *BRCA1/2* germline mutations or HR-deficiency [17]. In addition, clinical trials are ongoing to determine the benefit of combination of PARPi with other anti-cancer agents or epigenetic modulators (NCT03901469, NCT04508803).

## 10. Concluding Remarks and Future Perspectives

BRCA1 is a complex and multifaceted protein implicated in many important biological processes and plays a major role in homologous recombination-mediated DNA double-strand break repair. Although it is well known that *BRCA1* functions to maintain genome stability, it is now evident that *BRCA1* also plays an important role in cancer cell metastasis by regulating EMT, apicobasal polarity, and the tumor microenvironment. Genes implicated in these processes are likely viable targets to inhibit metastasis in *BRCA1* mutation-associated tumors; however, further investigation is required to determine specificity of these processes to *BRCA1*-deficient tumors considering the commonality in these fundamental processes across tumor subtypes. The various experimental and clinical studies discussed in this review have sought to determine TME changes induced by germline mutation in the *BRCA1* gene and how these changes impact tumor behavior and treatment response. In this review, we have attempted to provide meaningful insights into the role *BRCA1* plays in controlling cell invasion and metastasis, and the mechanisms by which the changes in TME could lead to breast cancer progression. Future studies should consider the interactions between tumor cells and their microenvironment, with the specific goal of improving cancer therapies for metastatic breast cancer patients.

Aforementioned, *BRCA1* mutations or loss-of-function have been linked to different cancers; however, historically, the vast majority of the studies have focused on tissues derived from breast and ovaries. Though this trend continues to this date, in recent years, an interest in examining the role of *BRCA1/2* and other DNA repair genes (i.e., ATM, MSH2, etc.) in metastatic prostate cancer and other cancer has emerged. In prostate cancer, *BRCA2* mutations are found at a significantly higher frequency of 24.3% compared to *BRCA1* mutations (6.4%) [95]. Mechanistically, *BRCA1* has been found to interact with the androgen receptor (AR) and functions as a coregulator to enhance AR transactivation in prostate cancer cells [96]. AR plays a pivotal role in prostate cancer [97]. Though androgen deprivation therapy can suppress most prostate cancers, a subset of high-risk tumors can progress to castration-resistant prostate cancers. In fact, AR aberrations are found in 62.7% of metastatic castration-resistant prostate cancers (mCRPC) [98]. An abundance of pre-clinical and clinical evidence implicates AR signaling in the development of both early and late-stage metastatic disease (reviewed in detail elsewhere [99]). *BRCA2* has been directly linked to prostate cancer metastasis. Loss of *BRCA2* has been demonstrated to promote prostate cancer invasion through up-regulation of matrix metalloproteinase 9 (MMP9) [100]. Indeed, functional *BRCA2* protein was found to limit the metastatic potential of cancer cells by downregulating MMP9 production via inhibition of the PI3K/AKT pathway and activation of the MAPK/ERK pathway, thus impairing migration and invasion of prostate cancer cells [100]. *BRCA1* (and BRCA2) have indisputable roles in maintaining genomic integrity in various cancers. At present, PARPi (specifically Olaparib and Rucaparib) have been approved for the treatment of mCRPC with germline *BRCA1/2* mutations [101]. Several clinical trials are currently underway to examine the efficacy of PARPi alone and in combination with other drugs in *BRCA1/2*-mutation carriers and in patients with tumors carrying mutations in other DNA repair genes in prostate and other cancers (NCT03148795, NCT04267939). Additional research is necessary to delineate the exact functions of these proteins outside of DNA repair in the development and progression of various cancers.

Herein, we have summarized recent advances in understanding the functions of *BRCA1* in DNA damage repair and breast cancer metastasis. We discussed the implications of *BRCA1/2* mutations in the course of breast cancer progression and metastatic recurrence, and also the therapeutic strategies used in the treatment of *BRCA1/2*-associated metastatic cancers. Understanding the role of *BRCA1/2* in tumor development, progression, and metastasis, will help determine the best course of action for patients with mutations in these genes and in patients with HR-defective DNA repair. Identification of metastasis-specific drivers from sequence analyses of biopsies from patients with metastatic tumors might aid in the development of personalized therapy. However, this process can be challenging considering that diversity in mutations may lead to different metastasis. Furthermore, collection of the biopsy samples for these studies poses additional challenges. Advances in the single cell sequencing technologies in combination with high resolution imaging techniques might help understand the processes underlying metastasis and assist in the discovery of druggable vulnerabilities that can suppress metastasis without systemic toxicity.
